# Atypical gaze patterns in children and adults with autism spectrum disorders dissociated from developmental changes in gaze behaviour

**DOI:** 10.1098/rspb.2010.0587

**Published:** 2010-05-19

**Authors:** Tamami Nakano, Kyoko Tanaka, Yuuki Endo, Yui Yamane, Takahiro Yamamoto, Yoshiaki Nakano, Haruhisa Ohta, Nobumasa Kato, Shigeru Kitazawa

**Affiliations:** 1 Department of Neurophysiology, Juntendo University School of Medicine, Tokyo, Japan; 3 Department of Pediatrics, Juntendo University School of Medicine, Tokyo, Japan; 2 CREST, JST, Saitama, Japan; 4 Japanese Institute for Education and Treatment, Tokyo, Japan; 5 Department of Psychiatry, Showa University School of Medicine, Tokyo, Japan

**Keywords:** eye tracking, eye movements, autism, development, mouth viewing, turn taking

## Abstract

Eye tracking has been used to investigate gaze behaviours in individuals with autism spectrum disorder (ASD). However, traditional analysis has yet to find behavioural characteristics shared by both children and adults with ASD. To distinguish core ASD gaze behaviours from those that change with development, we examined temporo-spatial gaze patterns in children and adults with and without ASD while they viewed video clips. We summarized the gaze patterns of 104 participants using multidimensional scaling so that participants with similar gaze patterns would cluster together in a two-dimensional plane. Control participants clustered in the centre, reflecting a standard gaze behaviour, whereas participants with ASD were distributed around the periphery. Moreover, children and adults were separated on the plane, thereby showing a clear effect of development on gaze behaviours. Post hoc frame-by-frame analyses revealed the following findings: (i) both ASD groups shifted their gaze away from a speaker earlier than the control groups; (ii) both ASD groups showed a particular preference for letters; and (iii) typical infants preferred to watch the mouth rather than the eyes during speech, a preference that reversed with development. These results highlight the importance of taking the effect of development into account when addressing gaze behaviours characteristic of ASD.

## 1. Introduction

Eye tracking has been used to investigate gaze behaviour in individuals with autism spectrum disorder (ASD) while viewing socially salient stimuli such as faces (Klin *et al*. 2002; Van der Geest *et al*. 2002; Dalton *et al*. 2005; von Hofsten *et al*. 2009). Fixation time analysis has been applied in most such studies so far, but the results have not been consistent (for a review, see Boraston & Blakemore 2007). For example, mouth-viewing data from adolescents and young adults with ASD reported in one study (Klin *et al*. 2002) were not reproduced when younger children served as participants (Van der Geest *et al*. 2002; von Hofsten *et al*. 2009). Von Hofsten *et al*. (2009) reported that not only do preschool children with ASD look at the mouth most of the time, but young children with typical development do as well. Thus, it has been suggested that the ratio of eye/mouth-viewing behaviours changes with development. To date, studies have yet to find characteristic gaze behaviours shared by both children and adults with ASD.

To distinguish core gaze behaviours in ASD patients from those that change with development, it is necessary to test both age groups using stimuli that can attract both groups. For this purpose, we examined temporo-spatial gaze patterns in young children and adults with and without ASD while they viewed identical short video clips taken from films and TV programmes for young children. Children were mental-age matched and adults were age matched. The clips featured one or more main characters who talked either to each other or to the audience watching the TV. Each scenario possessed varying degrees of social context and distracters so that socially and physically salient stimuli in the scene dynamically changed from one place to another, from one person to another, from the eyes to the mouth and from one moment to another within a fraction of a second.

To take into account all of the temporo-spatial gaze patterns from all of the participants, we summarized the data using multidimensional scaling (MDS). We were successful in finding two independent quantitative measures, of which one separated children from adults and the other separated participants with ASD from controls. Further post hoc analyses revealed gaze behaviours that characterized each group of participants; several of these characteristic gaze behaviours have not, to our knowledge, previously been reported.

## 2. Material and methods

### (a) Participants

There were four groups of participants, defined in a two-by-two factorial manner, which included children versus adults as well as those with and without ASD. A total of 104 participants participated in the study. The study received approval from the institutional ethics committee, and all participants or parents of child participants gave written informed consent according to institutional guidelines.

### (b) Children with ASD

Twenty-five children (21 boys and four girls) with ASD participated. Twenty met the criteria to be diagnosed with autistic disorder, and five were diagnosed with pervasive developmental disorder-not otherwise specified (PDD-NOS) according to the DSM-IV (American Psychiatric Association 2000). Diagnoses were established based on the clinical judgement of medical specialists. The mean chronological age of these children was 4∶11 years and ranged between 2∶8 and 9∶0 years. The developmental evaluation was carried out with the Bayley Scales of Infant Development, 2nd edition up to the developmental age of 3∶6 years (42 months); the Japanese Kaufman Assessment Battery for Children was used thereafter. These results are shown in electronic supplementary material, table S1. The mean developmental age was 3∶1 years (range: 1∶4–7∶6 years; s.d.: 1∶10 years). Two additional children were tested but excluded from the analysis because they could not remain seated on the chair.

### (c) Typical children

Twenty-five typically developing (TD) children (14 boys and 11 girls) participated as controls, with a mean age of 3∶1 years (range: 1∶2–7∶9 years; s.d.: 1∶11 years). Their chronological ages were matched to the developmental age of the children with ASD (11 1-year olds, four 2-year olds, four 3-year olds, two 4-year olds, three 6-year olds and one 7-year old). The TD children did not have a family history of ASD. Two additional children were tested but excluded from the analysis because their total viewing time did not reach a cut-off of 35 s.

### (d) Adults with ASD

Twenty-seven adults (18 males and 9 females) with ASD participated, with a mean age of 29.5 years (s.d.: 7.4 years). According to DSM-IV criteria, this group comprised seven with autistic disorder, 16 with Asperger syndrome and four with PDD-NOS. ASD diagnoses were established based on the clinical judgement of two medical specialists. All participants were highly functioning such that their full-scale intelligence quotient (IQ) (Wechsler Adult Intelligence Scale, 3rd edn, WAIS-III) exceeded 75 (mean = 104, electronic supplementary material, table S2). All participants answered questionnaires for the Japanese version of the autism spectrum quotient (AQ) test (Baron-Cohen *et al*. 2001). Among the 27 adults with ASD, six were taking one or more medications; five were taking antidepressant drugs, two were taking tranquilizers, two were taking antipsychotic drugs and two were taking antiepileptic drugs. One additional participant was tested but excluded from the analysis because the data were unstable.

### (e) Typical adults

Twenty-seven typical adults (16 males and 11 females) participated as controls, with a mean age of 32.1 years (s.d.: 11.8 years). The typical participants did not have a family history of ASD and were free from any history of psychiatric disorders.

### (f) Apparatus

Gaze positions of both eyes were measured at 50 Hz with a remote eye tracker (Tobii, ×50, Tobii Technology AB). A box with infrared sources and a camera were set below a 17-inch TFT flat-screen monitor (Eizo, FlexScan S1701, full screen was used at a resolution of 640 × 480 pixels). Participants were seated on a chair facing the screen, which was at a distance of 60 cm from their eyes. Children under 3 years old were seated on the lap of a parent. The accuracy of the eye tracker was 0.5° of the visual angle, which corresponded to 0.5 cm and 10 pixels on the screen. The device had a tolerance for head motion of 30 cm horizontally, 16 cm vertically and 20 cm in depth.

### (g) Stimuli

The video stimulus was 77 s long and consisted of 12 short video clips with sound, each of which lasted for approximately 6 s. Each video clip was taken from a film or TV programme for young children and involved one, two, three or more human characters who were either talking to the audience or talking to each other (electronic supplementary material, figure S1).

### (h) Data analysis

Gaze positions of the right and the left eyes were averaged to yield a single gaze position for each time point. Data points were included for further analysis only when both eye positions were available. The results that included valid data points for at least 35 s were used for further analysis. The total viewing times (lengths of time with valid data points, mean ± s.d.) were 70 ± 8 s for TD adults, 72 ± 5 s for TD children, 70 ± 9 s for ASD adults and 57 ± 12 s for ASD children. Total viewing time for ASD children was significantly shorter than for TD children mostly owing to recording errors resulting from gradual changes in posture (see electronic supplementary material, figure S5 for details).

### (i) Full gaze pattern analysis

To quantify differences and similarities in the temporal pattern of gaze movements among participants, we directly calculated the absolute distance between every pair of gaze points at each 50 Hz timepoint. If the two participants looked at the same point in the screen, the distance is zero. If the two participants looked 200 pixels apart, the distance is 200. The distance was calculated at each of 3850 time points (50 Hz × 77 s) and the median was taken as the distance between the two participants. This procedure was repeated for every participant pair to define a between-participant distance matrix (104 × 104). With this matrix, we applied MDS (Kruskal & Wish 1978) to plot each participant in a two-dimensional plane (MDS plane).

If there are only three participants, the distances between three pairs determine a triangle with each participant at each apex of the triangle on a two-dimensional plane. To represent exact distance between every pair of 104 participants, we need a 103 dimensional space. By using MDS, we determined the position of each subject on a two-dimensional plane so that the distance between each pair was conserved as much as possible. We used Matlab statistics toolbox (mathworks) for calculations.

If the temporo-spatial gaze trajectories were similar for a pair of participants, they would be plotted very near each other and vice versa. Thus, a group of participants with similar gaze behaviours would form a cluster in the plane, whereas those with atypical gaze behaviours would be plotted towards the periphery and far from the other participants.

The distance from the median for the entire distribution in the MDS plane (MDS distance) was taken as the distance from a standard gaze behaviour. The MDS distance was compared across groups using one-way analysis of variance (ANOVA) and a post hoc analysis (Ryan's method, Day & Quinn 1989). Receiver-operating characteristic (ROC) curves were used to define an MDS distance threshold for distinguishing the ASD groups from the TD control groups. We further examined whether we were able to discriminate the child groups from the adult groups in the MDS plane by using a linear discriminant analysis.

### (j) Frame-by-frame analysis

We then analysed each frame of the video clips to clarify the cause of across-group differences revealed by the full trajectory analysis. For this purpose, we invented a new method that automatically assigned a continuous value from zero to one according to the distance between the gaze position and each target. We first identified major targets, including eyes, nose, mouth and ears of all faces, by hand in each of 2237 frames (see electronic supplementary material, figure S2 for details). Then, the distance between a gaze position and the registered target was calculated, and a value from zero to one was assigned using a Gaussian function.

### (k) Viewing proportion analysis

To quantify how many participants viewed a particular target (e.g. eyes or mouth of a particular character or the face of one boy versus that of another boy), we calculated the sum of the assigned values in a participant group and divided the sum by the number of participants in that group. The viewing proportion for a particular target was plotted against the time for each group in order to find the group difference.

To compare gaze durations for the eyes and mouth, three video clips with facial close-ups (clips 3, 7 and 11 in electronic supplementary material, figure S1) were chosen. Sums of the assigned values were divided by the total duration of the gaze time so that the viewing times for the eyes and mouth were quantified as percentages. Per cent viewing times for the eyes, nose, mouth and ears were summed to yield the total viewing time for the face.

## 3. Results

### (a) Full gaze pattern analysis

**Figure 1.**
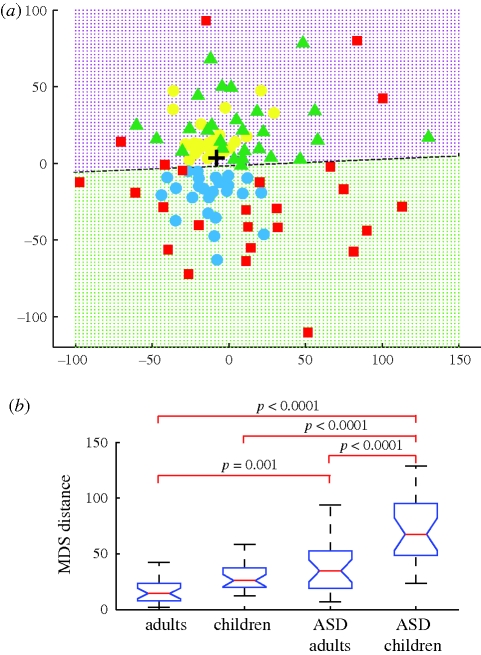
Temporo-spatial gaze patterns quantified by the full trajectory analysis using MDS. (*a*) Distribution of gaze patterns in the MDS plane. Each symbol represents a full gaze trajectory (3728 gaze positions) from a single participant who belonged to the typical adult group (yellow circles), typical child group (light blue circles), adults with ASD group (green triangles) or children with ASD group (red squares). A black cross indicates the median position of the entire distribution. The broken line discriminates adults (purple dotted area) from children (green dotted area) and was calculated using a linear discriminant analysis. (*b*) Group comparison of the MDS distance measured from the cross in (*a*). On each box, the central mark is the median, the edges of the box are the 25th and 75th percentiles and the whiskers extend to the most extreme data points that were not considered outliers if they fell within 1.5 times the box length. Notches in each box show a 95% confidence interval of the median.

In the MDS plane, TD children (light blue circles) and typical adults (yellow circles) were distributed near the centre of the plane (figure 1*a*). This indicates that the typical participants were similar in their gaze patterns. In marked contrast, children with ASD (red squares) and adults with ASD (green triangles) were distributed towards the periphery. We regarded the median of the entire distribution (cross), which fell in the centre of the typical participants, as representing the standard temporo-spatial gaze pattern. Then the distance from the standard (the MDS distance) would be smaller in the control groups than in the ASD groups. A one-way ANOVA revealed a significant main effect of group type (*F* = 21.1, *p* < 0.00001), and post hoc analysis showed significant group differences between the children with ASD and the other groups, as well as between adults with ASD and adults with typical development (figure 1*b*; Ryan's *Q* test; see figure 1*b* for *p*-values). These results show that typical control groups share similar temporo-spatial gaze patterns, whereas those with ASD show atypical gaze behaviours that are different from one participant to another.

We further evaluated how well the MDS distance distinguished individuals with ASD from typical controls using ROC curves. The areas under the ROC curves indicated that the discrimination level was 0.75 (0.5 for no discrimination and 1.0 for perfect discrimination) for adults and 0.87 for children. Both exceeded a threshold for an acceptable level of discrimination (0.7) and that for children (0.87) exceeded a threshold for excellent discrimination (0.8) (Hosmer & Lemeshow 2000). The MDS distance did not significantly correlate with the total viewing time in any of the four groups (*r* = 0.20, 0.32, 0.11, 0.16; *p* = 0.33, 0.12, 0.57, 0.46). Further analysis of the adults with ASD showed that the MDS distance correlated with the AQ (electronic supplementary material, figure S3*a*) but not with the IQ (WISC-III, electronic supplementary material, figure S3*b*–*d*), thereby demonstrating that the MDS distance was a good indicator of core symptoms associated with ASD.

It is also worth noting that both adult groups were distributed in the upper half of the plane, whereas both child groups were in the lower half. A dotted line near the *x*-axis was calculated by applying a linear discriminant analysis; this line distinguished children from adults with an accuracy of more than 95 per cent. All adults (54/54 = 100%) were plotted above the line, and nearly all children (except for five children with ASD) were plotted below the line (45/50 = 90%). In other words, temporo-spatial gaze patterns in typical children appear to develop into adult gaze patterns along the *y*-axis. Although gaze patterns in individuals with ASD were highly variable, as was revealed by their wide distribution along the periphery of the MDS plane, these individuals also share mature gaze patterns generally observed in control individuals with typical development.

### (b) Eye/mouth-viewing proportion analysis

**Figure 2.**
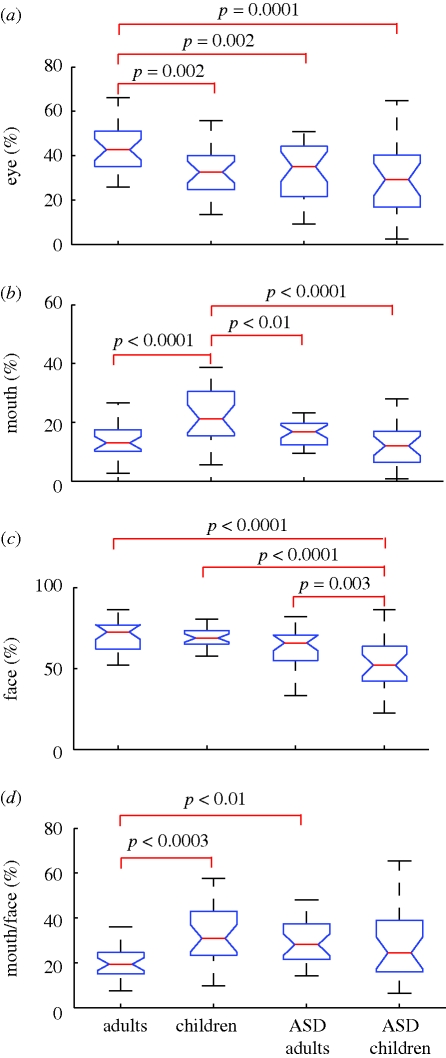
Group comparisons of viewing proportions for the (*a*) eyes, (*b*) mouth, and (*c*) face, and (*d*) the mouth-viewing proportion normalized by the face-viewing proportion. One-way ANOVA revealed that the main effect of the group was significant for the eye-viewing proportion (*F*_3,100_ = 6.4, *p* = 0.0005), mouth-viewing proportion (*F*_3,100_ = 9.7, *p* < 0.0001), face-viewing proportion (*F*_3,100_ = 11.9, *p* < 0.0001), and mouth-viewing proportion normalized by the face-viewing proportion (*F*_3,100_ = 4.5, *p* < 0.006). Brackets show the pairs that yielded significant differences in post hoc analyses (Ryan's *Q* test).

Next, we examined whether the clear separation of the four groups in the MDS plane was also reflected in differences in the viewing time of the eyes, mouth and face as a whole, because impairments in processing of faces have been implicated in persons with ASD (Langdell 1978; Klin *et al*. 2002; Jemel *et al*. 2006). One-way ANOVA and post hoc tests revealed four principal findings. First, the eye-viewing proportion relative to the total viewing time was longest in typical adults (mean = 44%) and significantly longer than in the other three groups (figure 2*a*), i.e. adults with ASD (33%), typical children (33%) and children with ASD (30%). It is worth noting that there was no significant difference between children with ASD and typical children.

Second, the mouth-viewing proportion was longest in typical children (23%) and significantly longer than in the other three groups (figure 2*b*), which included children with ASD (13%), typical adults (14%) and adults with ASD (17%). Based on a previous study (Klin *et al*. 2002), we expected that individuals with ASD would look at the mouth longer than typical controls would (at least among adults). Indeed, the mean mouth-viewing proportion was longer in adults with ASD (17%) than in typical adults (14%), but the difference was not significant. Interestingly, this difference was completely reversed in children (typical 23% > ASD 13%; *p* < 0.0001).

Third, face-viewing proportions were significantly shorter in children with ASD (51%) than in the other three groups (figure 2*c*), i.e. typical adults (71%), typical children (69%) and adults with ASD (62%). However, the difference between adults with ASD and typical controls was not significant.

Fourth, the mouth-viewing proportion relative to the face-viewing time was significantly longer in typical children (61%) than in typical adults (48%) and adults with ASD (50%). However, no significant difference was observed when comparing typical children with children with ASD (55%). Thus, there was no significant difference between child groups (figure 2*d*).

Each of the viewing proportions showed a significant difference between adult groups (eye, eye/face; typical > ASD) and between child groups (mouth, face; typical > ASD), but none showed differences common to both age groups.

### (c) Frame-by-frame analysis

**Figure 3.**
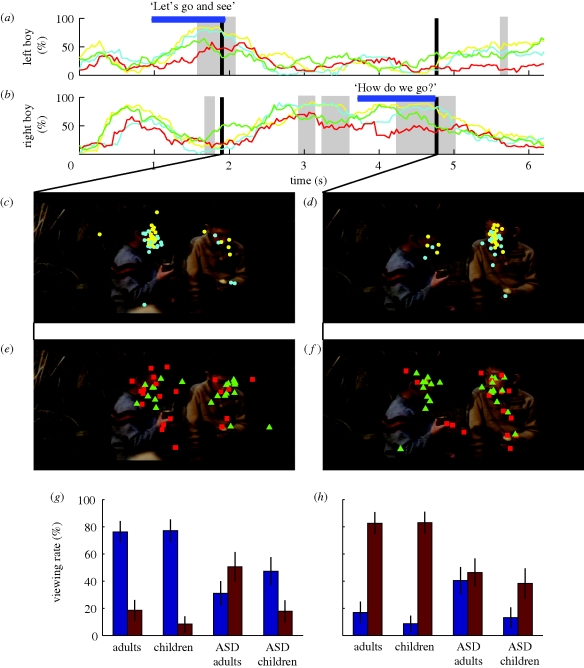
Frame-by-frame analysis of clip 4, in which two boys took turns in a dialogue. (*a*,*b*) Temporal profiles of the viewing proportion for the (*a*) right and (*b*) left boy. (*a*,*b*) Yellow, adults; cyan, children; green, ASD adults; red, ASD children. Each horizontal bar indicates the period during which each boy delivered his dialogue (shown on the bar). Shaded areas show periods of significant difference in the viewing proportions between control and ASD groups (Wilcoxon rank-sum test: *p* < 0.01, uncorrected). (*c–f*) Gaze plots of typical adults (yellow circles) and children (light blue circles) at (*c*) 1.9 s and (*d*) 4.8 s, and of adults and children with ASD (green triangles and red squares) at (*e*) 1.9 s and (*f*) 4.8 s. (*g*,*h*) Group comparisons of face-viewing proportions for the right (brown) and left (dark blue) boys at (*g*) 1.9 s and (*h*) 4.8 s. Error bars show the s.e. of the mean.

To further identify which part of the video clip was responsible for the clear between-group difference in distributions along the MDS plane, we drew temporal profiles of the viewing proportion for each target and for each group and carried out frame-by-frame comparisons (e.g. figure 3*a*,*b*). Here, we describe the three video clips (clips 4, 5 and 11, electronic supplementary material, figure S1) that yielded the largest number of frames with significant differences in the viewing proportion between participants with and without ASD (clips 4 and 11), as well as between children and adults (clips 5 and 11).

#### (i) Clip 4

In clip 4 (6 s long), two boys talked in turn. At first, most control participants (children, cyan; adults, yellow) viewed the boy on the right (from 0.8 to 1 s), who showed his full face to the audience with his eyes looking down. Then, most gazes shifted to the boy on the left and reached a peak viewing proportion of 90 per cent as he said ‘Let's go and see’ at 1.9 s (note the peak in figure 3*a* and the gaze plots in figure 3*c*). As soon as he finished talking, gazes shifted again to the boy on the right (figure 3*b*). The participants kept looking at the boy on the right for about 2 s, until at last he uttered the phrase ‘How do we go?’ at 4.8 s (figure 3*d*).

In contrast to the dynamic shifts of gaze shared among control participants, the peaks in face-viewing proportions were relatively low in adults and children with ASD (green and red lines, respectively, in figure 3*a*,*b*). At 1.9 s, for example, the gaze positions of adults and children with ASD were widely scattered (figure 3*e*). This scattering was in marked contrast to the gazes of the control participants, who concentrated on the face of the boy on the left (figure 3*c*). Some children with ASD (red squares) looked at the sweet potato, and the majority of adults with ASD (green triangles) focused their gazes on the boy on the right instead of the boy on the left (figure 3*e*,*g*). At 4.8 s, when most control participants looked at the face of the boy on the right (figure 3*d*), adults and children with ASD looked broadly over his face and body (figure 3*f*). These different gaze behaviours at 1.9 and 4.8 s are summarized in two histograms (figure 3*g*,*h*).

#### (ii) Clip 11

In clip 11 (5.5 s in duration), a young girl began to appear at 0.9 s as curtains started to open, and her full face became visible at 2 s. Then, from 3.1 to 4.8 s, she announced her name. Simultaneously, a caption showing the girl's name and age appeared at the bottom of the screen until the end of the video clip. In this clip, we found differences not only between typical controls and ASD participants but also between children and adults.

**Figure 4.**
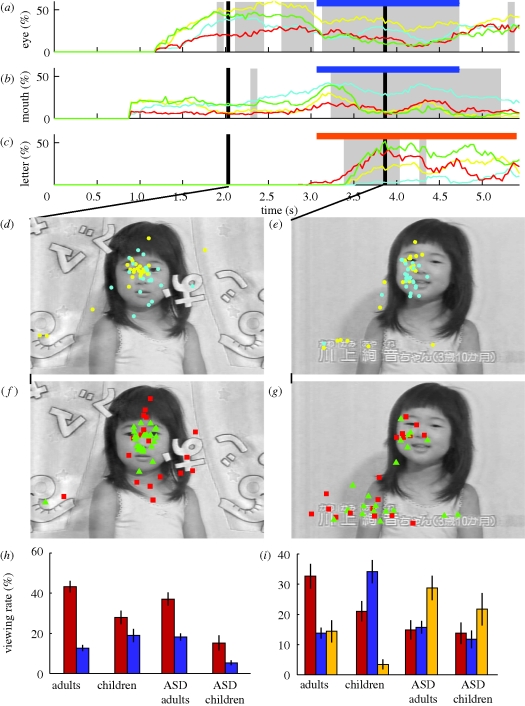
Frame-by-frame analysis of clip 11, which featured a girl who announced her name. (*a–c*) Temporal profiles of the viewing proportion for the (*a*) eyes, (*b*) mouth and (*c*) letters. (*a*–*c*) Yellow, adults; cyan, children; green, ASD adults; red, ASD children. A short horizontal bar indicates the period during which the girl announced her name (*a*,*b*) and the long bar in (*c*) indicates the period during which her name was shown by a caption at the bottom of the screen. The shaded areas show periods with significant differences in the viewing proportions between control and ASD groups. (*d–g*) Gaze plots of typical adults and children (yellow and light blue circles) at (*d*) 2.0 s and (*e*) 3.9 s and of adults and children with ASD (green triangles and red squares) at (*f*) 2.0 s and (*g*) 3.9 s. (*h*,*i*) Group comparisons of the viewing proportions for the eyes (red) and mouth (dark blue) at (*h*) 2.0 s and (*i*) 3.9 s. The viewing proportions for the letters (yellow columns) are also shown in (*i*).

Typical adults generally looked at the eyes of the girl as soon as her eyes became visible at 1.8 s and thereafter (figure 4*a*, yellow curve; figure 4*d*).

Adults with ASD were similarly quick to look at the eyes of the girl at 2 s (figure 4*f*, green dots) and looked briefly at the mouth when she began speaking at 3.1 s (figure 4*b*). However, when the caption appeared, these adults generally stopped looking at the girl and shifted their gazes to the caption (figure 4*c*,*g*) for more than 1 s, which was much longer than required to read the caption.

Typical children (figure 4*a*, cyan) were slow in their initial saccade to the eyes of the girl compared with typical adults (yellow) and adults with ASD (green), but typical children looked more at the eyes than at the mouth at 2 s. However, typical children gradually shifted their gazes to the mouth and kept looking at the mouth throughout the period when the girl announced her name (blue bars, 3.1–4.8 s, figure 4*b*); furthermore, they almost ignored the caption (figure 4*c*, cyan).

The gazes of children with ASD (red) were widely scattered compared with the gazes of the other groups. The gazes of children with ASD were initially drawn to moving objects, which included the curtain and lettering, and were scattered around the neck of the girl (figure 4*f*). However, their gazes were drawn to the caption even more quickly than in adults with ASD (figure 4*f*,*g*). This was unexpected because most of the children with ASD could not read letters, as was the case with the typical children. These different gaze behaviours at 2.0 and 3.9 s are summarized in two histograms (figure 4*h*,*i*).

#### (iii) Clip 5

In this video clip, a male gymnastics instructor instructed the audience watching the TV (electronic supplementary material, figure S4*c*). This clip yielded two typical differences between children and adults.

First, children were generally slower than adults in their first saccades to the face of the main character in the scene (electronic supplementary material, figure S4*a*,*c*,*e*,*g*; at 0.4 s). Second, compared with adult participants, children showed a strong preference for animal characters (electronic supplementary material, figure S4*b*,*d*,*f*,*h*; at 3.8 s).

## 4. Discussion

In the present study, we examined temporal as well as spatial gaze patterns of children and adults with and without ASD while they viewed ecologically relevant video stimuli. By using MDS that took all temporo-spatial gaze trajectories into account, we quantitatively demonstrated that typical participants shared highly stereotypical gaze patterns, whereas individuals with ASD were variable in their gaze patterns. In addition, we found a clear separation between children and adults in the two-dimensional MDS plane. Further post hoc frame-by-frame analyses revealed several gaze behaviours that characterized each group of participants, although we admit that the targets of the post hoc analysis did not cover all possibilities underlying the group differences in the MDS analysis. In fact, we focused mainly on the gaze behaviours towards face parts in the post hoc analyses because impairments in the processing of faces have been implicated in persons with ASD (Langdell 1978; Klin *et al*. 2002; Jemel *et al*. 2006).

### (a) Shorter fixation on the face in ASD groups

A typical difference between ASD groups and typical control groups appeared while they viewed a video clip showing two boys taking turns speaking in a conversation (clip 3, figure 3). Both TD children and TD adults fixated on the face of each character until the end of his speech. By contrast, children and adults with ASD did not keep their gaze fixed until the end of the boy's speech. As a result, the greatest difference in the viewing proportion occurred at the end of each speech (1.9 s for the left boy and 4.8 s for the right boy, figure 3).

Von Hofsten *et al*. (2009) reported that children with ASD (mean age of 4.8 years) made predictive saccades from one speaker to the next less often than 3-year-old control participants. In agreement with this result, we found that children with ASD made fewer gaze shifts than typical children during a period of predictive saccades (time: 1.9–2.7 s, figure 3*b*) from the first speaker (boy on the left) to the second (boy on the right).

It is worth noting that typical adults were rather conservative in making predictive saccades. They seemed to have made a saccade to the next speaker only when they were sure that the first one had finished his speech. By contrast, adults with ASD made premature saccades to the next speaker while the first speaker was still speaking. Thus, the lack of predictive saccades may apply to children with ASD but not to adult participants. Rather, it is an early shift of gaze away from the speaker in charge that characterized ASD participants in both age groups.

### (b) Preference for letters in ASD groups

Another difference was observed when a caption appeared while the main character announced her name (figure 4, clip 11). It was natural for typical children not to shift their gazes to the caption because most of them were too young to read. However, both adults and children with ASD (some of whom were impaired even in their speech) showed a strong preference for the letters in the caption (figure 4*g*). This was in marked contrast to typical children and adults, who fixated mostly on the face of the girl who was saying her name (figure 4*e*). This finding generally agrees with previous studies that reported a preference in ASD participants for non-human objects over human faces (Klin *et al*. 2002), as well as a preference for non-human sounds over human voices (Gervais *et al*. 2004), a better perception of objects than the eye as revealed in slit viewing in which participants named an object that moved behind a narrow slit (Nakano *et al*. 2009) and larger brain event-related potentials to objects (Webb *et al*. 2006) than in typical controls.

### (c) Mouth-viewing behaviour in typical children

A striking difference between typical children and typical adults appeared when they looked at the face of the girl who announced her name (figure 4*e*). The majority of typical children looked at the mouth, whereas the typical adults kept looking at the eyes (figure 4*b*). This tendency was confirmed by our analysis of three video clips that involved facial close-ups (figure 2*b*). We inferred from this result that viewing the mouth of a person during speech is a necessary step in typical development and is a characteristic that disappears as the child grows into adulthood. This view agrees with a recent finding indicating that greater amounts of fixation time on the mother's mouth during live interactions at the age of six months predicted higher levels of expressive language at the age of 18 months (Young *et al*. 2009).

These results in typical children agree with a study by von Hofsten *et al*. (2009) that showed clear mouth-viewing behaviours in both 1-year-old (94% of the time) and 3-year-old children (99%) with typical development. However, they also reported comparable mouth-viewing behaviours in children with ASD (96% of the time), although the mouth-viewing proportion of ASD children was relatively lower in the present study than in the study by von Hofsten *et al*. This result was probably owing to the numerous distracters in our stimuli, which included letters in the caption, as well as heads and body parts that moved, in contrast to the stimuli used by von Hofsten *et al*. (2009).

In a recent study, Jones *et al*. (2008) reported that 2-year-old toddlers with ASD looked at the mouth, whereas their matched control group looked at the eyes, when viewing videos that featured an actress looking directly into the camera while playing pat-a-cake (or other activities). This finding seems to contradict our results and those of von Hofsten *et al*. (2009); hence, we have pointed out two factors that could explain these discrepancies. First, the actress in the video clapped her hands below the mouth (e.g. fig. 2 in Jones *et al*. 2008). These same researchers recently reported that toddlers with ASD showed a strong preference for contingencies of visual stimuli and sounds that typically occurred with the hands when playing pat-a-cake (Klin *et al*. 2009). Thus, the hands inevitably drew the attention of the toddlers with ASD to the lower half of the screen, where both the hands and the mouth were located. Second, in our study and in the study by von Hofsten *et al*. (2009), the main characters spoke names or sentences that the children were hearing for the first time, but the actress in Jones *et al.* (2008) was singing a song familiar to typical toddlers. This might have reduced the importance of the mouth in collecting information that helps hearing. In fact, typical children in our study also looked initially at the eyes of the girl when she was silent.

Klin *et al*. (2002) reported that adolescents and young adults with ASD (15 years old on average) spent more time viewing the mouth than typical controls (18 years old on average). Our findings agree with this result (ASD > typical) as far as the adult participants are concerned. However, our results for children were the opposite (typical > ASD). This reversal of mouth-viewing time from childhood (typical > ASD) to adulthood (ASD > typical) explains the variability among previous studies examining eye/mouth-viewing behaviours in participants with and without ASD (Klin *et al*. 2002; Van der Geest *et al*. 2002; Boraston & Blakemore 2007; von Hofsten *et al*. 2009). Thus, longer mouth viewing in ASD adults may be a kind of compensation that is acquired after childhood (Klin *et al*. 2002; Norbury *et al*. 2009).

### (d) Clinical implications of the MDS distance

The distance from the ‘centre’ of the MDS plane, which reflected the degree of deviation from a standard temporo-spatial gaze pattern, efficiently distinguished control participants from those with ASD in both children and adults. The MDS distance in adults correlated with the AQ (Baron-Cohen *et al*. 2001) but not with verbal IQ or performance IQ, thereby suggesting that the MDS distance reflected (at least in part) core deficits in ASD.

As discussed, eye/mouth-viewing proportions cannot discriminate typical participants from those with ASD in either children or adults, owing to a dramatic change in mouth-viewing behaviour during the course of typical development. Recent studies examining gaze behaviours have reported significant group differences in the target of the first saccades (Fletcher-Watson *et al*. 2009; Senju *et al*. 2009) and in fixation times for upright and inverted videos featuring biological motion (Klin *et al*. 2009). However, none of these tests has been applied to both children and adults. Thus, the present method, which used a 77 s long video in combination with the MDS distance, was unique in that it used a quantitative method applicable to both child and adult participants.
